# Impact of a randomized controlled trial of discounts on fruits, vegetables, and noncaloric beverages in NYC supermarkets on food intake and health risk factors

**DOI:** 10.1371/journal.pone.0291770

**Published:** 2023-11-22

**Authors:** Aniema Nzesi, Benedicta Owusu, Jillian Barry, Manveer Sandhu, Allan Geliebter

**Affiliations:** Department of Psychiatry, Mount Sinai Morningside, Icahn School of Medicine at Mount Sinai, New York, New York, United States of America; Federal University of Minas Gerais: Universidade Federal de Minas Gerais, BRAZIL

## Abstract

The objective of this study was to observe the effects of a multi-level (30%, 15%, and 0%) randomized discount on fruits, vegetables, and non-caloric beverages on changes in dietary intake. This randomized controlled trial (RCT) comprised an 8-week baseline, a 32-week intervention, and a 16-week follow-up. 24-hour dietary recalls were conducted during the baseline period and before the intervention midpoint. In-person clinical measures were analyzed from Week 8 (end of baseline) and 24 (midpoint). This report is from an interim analysis up to the intervention period midpoint at Week 24, as the study is still ongoing. Participants with BMIs of 24.5–50 kg/m^2^ and ages 18–70 years old who were the primary household shoppers were recruited from several New York City supermarkets, starting in September 2018. Of these, we analyzed 20 in the 30% discount group, 25 in the 15% discount group, and 19 in the 0% discount group. The 30% discount group reported greater intake of vegetables (+98.4 g ± 48.9 SD, *P* = 0.049) and diet soda (+63.3 g ± 29.3, *P* = 0.035) relative to the baseline period, compared to the 0% discount group. The clinical measures including body weight remained unchanged. The participants who experienced the COVID-19 pandemic had a marginal increase in body weight of 1.5 kg, P = 0.053. In conclusion, we observed a significant increase in intake of vegetables and diet soda in the 30% discount group relative to the 0% discount group.

## Introduction

Dietary food intake has a major influence on health indicators, including Body Mass Index (BMI), blood pressure, serum cholesterol and glucose [1]. Intake of sugar-sweetened beverages (vs. non-caloric beverages), the primary sources of added sugars, can also affect BMI [2]. Decisions to purchase specific food items are primarily based on taste and cost [3, 4]. Affordability of food items is a limiting factor for meeting fruit and vegetable intake guidelines. In the U.S., only 12% of adults met fruit recommendations and 10% met vegetable recommendations [5]. More affordable low energy-dense (ED) foods, such as fruit and vegetables, could lead to their increased intake [4].

The main source of food for homes in the U.S. is the supermarket [6]; however, cost is a limiting factor in purchasing, especially for nutrient-dense, low ED foods such as fruits and vegetables, which are relatively more expensive than high ED foods [7, 8]. There are papers examining the effects of price-related interventions on purchasing of healthy foods, but only a few look at dietary intake of fruits and vegetables [9]. A randomized controlled trial (RCT) study that employed a 50% discount on fruit and vegetables noted a 50% increase in consumption of these food items [10]. In another U.S. study, the intervention group received $1 for every healthy food/beverage purchased, based on the submission of store receipts and food labels. The intervention group significantly increased vegetable intake obtained from 3-day food records [11]. In a third study, Supplemental Nutrition Assistance Program (SNAP) participants were randomly assigned to receive a 30% incentive (vs. no incentive) on fruits and vegetables [12]. There was a significant increase in fruit and vegetable intake (via 24-h dietary recalls) in the treatment group compared to the control group.

The purpose of this study was to investigate the effects of multi-level supermarket discounts at the point of purchase on dietary intake and clinical markers of health. Economic interventions that aim to make healthful foods more affordable can result in changes in dietary intake behaviors and health outcomes. This is the first RCT to systematically examine the relatively long-term impact of multilevel discounts of fruits, vegetables, and non-caloric beverages on dietary intake (based on unannounced 3-day 24-hour dietary recalls) and clinical health markers, including body weight, percent body fat, blood pressure, fasting serum glucose, hemoglobin A1C, and serum blood lipids in a real world urban supermarket setting. The inclusion of multiple discount levels can aid selection of ideal discounts. Furthermore, the intervention employs electronic store loyalty cards, providing a novel method of issuing price discounts on fruits, vegetable, and non-caloric beverages at the point-of-sale. We predicted that fruit, vegetable, and non-caloric beverage intake (primary outcomes) would increase in the 30% (primary intervention) and 15% discount groups compared to the 0% discount (control) group. Additionally, we predicted decreases in the secondary outcomes of body weight (kg), blood pressure (mmHg), total cholesterol (mg/dL), HDL- and LDL-cholesterol (mg/dL), triglycerides (mg/dL), hemoglobin A1c (%), and fasting serum glucose (mg/dL) in the intervention groups. Because the study period overlapped with the COVID-19 pandemic, we also examined the effect of COVID-19 on body weight and other health risk factors, and we entered COVID-19 as a covariate for the primary and secondary outcomes.

## Materials and methods

### Study design

In the current RCT, qualified supermarket shoppers with overweight or obese status were allocated to receive a 30%, 15%, or 0% (control) discount on fruits, vegetables, and non-caloric beverages. The study had an 8-week baseline period, a 32-week intervention period, and a 16-week follow-up period. Hospital visits took place at weeks 0, 8, 24 (halfway during the intervention), 40 (end of intervention) and 56. As the study is still ongoing and undergoing data checking and analysis, we report the interim results for those who reached the halfway mark of the intervention period at week 24. The length of the study, 13-months for each participant, and a 7-month hospital-mandated hiatus due to the COVID-19 pandemic from March 2020 to November 2020, during which there was an increase in drop-out rate, raised a concern that there would be fewer participants completing the study than the number who reached the midpoint. All these reasons made it worthwhile to report the interim findings. This study was conducted according to the guidelines in the Declaration of Helsinki and all procedures involving research study participants were approved by the Icahn School of Medicine Institutional Review Board. Written informed consent was obtained from all participants. As a randomized controlled trial, this study was registered with *clinicaltrials*.*gov*. All participants signed consent forms during an initial screening visit.

### Sample size

Sample size calculation employed G*Power (3.1.9.7) for the primary outcome variable of fruit and vegetable intake. It was based on a similar RCT conducted using a 50% discount over 8 weeks vs 0% discount [10], which we assumed would yield a similar impact as a 30% discount over 16 weeks in the current study. In the previous study, there was a significant difference between the baseline and intervention periods for the two groups, effect size *d* = 0.85. For power = 0.80, 1-tailed α = 0.05, a minimum *n* = 18 per group would be needed. Thus, our mean sample size per group *n* = 21 was considered adequate.

### Participants

From September 2018 to April 2021, study participants were recruited on a rolling basis from two Foodtown, two Super Foodtown and two Brooklyn Harvest supermarkets in Manhattan, Brooklyn, and Queens, all associated with Allegiance Retail Services and carrying the same food items. Shoppers were recruited in the stores by research assistants standing next to posters and by advertisements posted in the store and on store receipts. Study participants were compensated $55.50 for the initial medical screening visit, which included $5.50 to cover a round trip Metropolitan Transit Authority (MTA) fare, and $90.50 for subsequent visits, which also included a round trip MTA fare.

**Fig 1:**
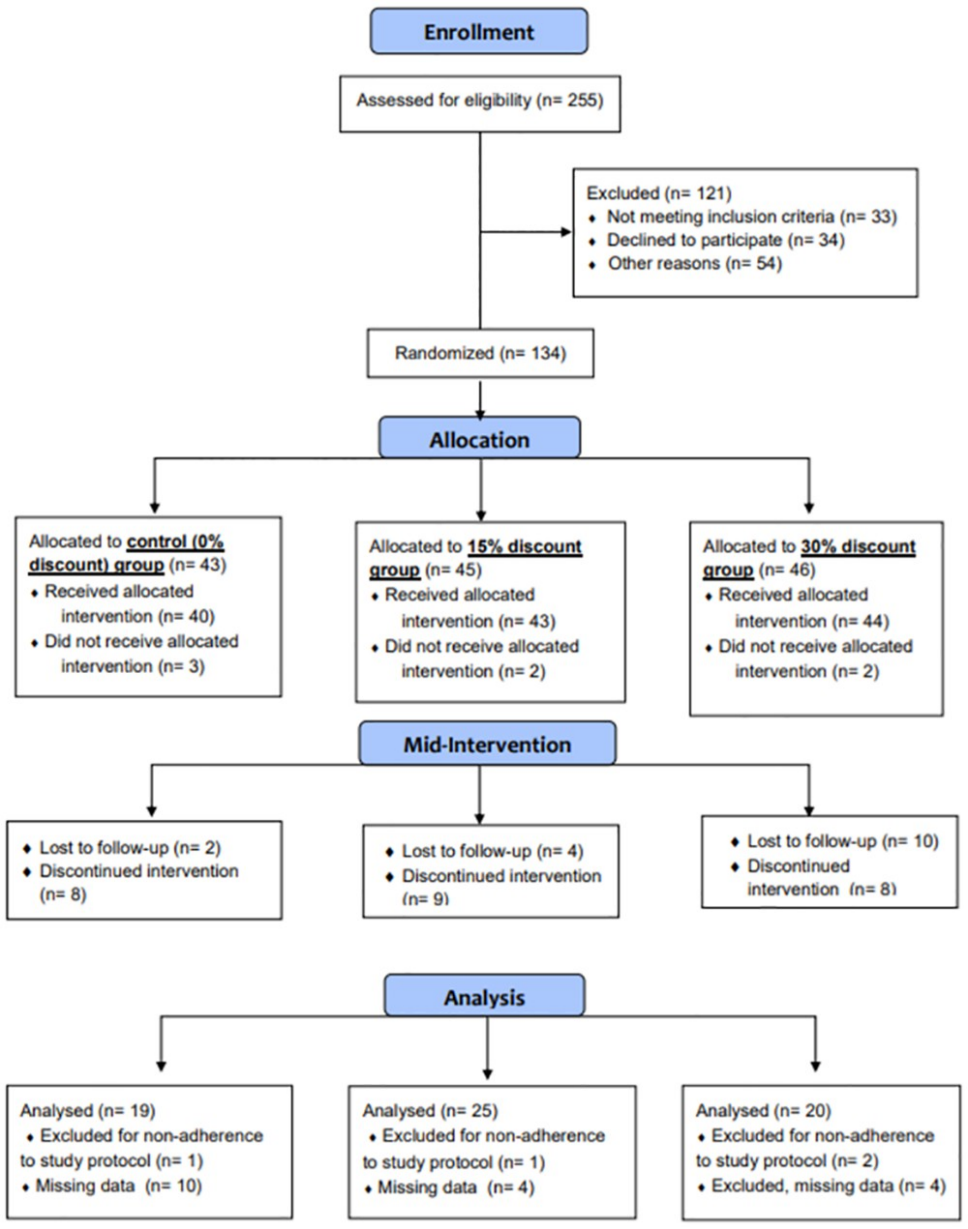
CONSORT diagram.

Of the 255 shoppers screened for eligibility, 134 participants were enrolled and randomized into one of the intervention groups. The other 121 participants either did not meet the inclusion criteria, dropped out of the study prior to randomization, or were withdrawn from the study. Of the randomized participants, 7 dropped out or were withdrawn prior to receiving the intervention and 38 after receiving the intervention. The attrition rate up to the study midpoint was 34% (45/134). Dropouts and withdrawals were largely due to the COVID-19 pandemic and included: moving from the area, a 7-month institutional study pause due to the pandemic, discomfort with returning to the hospital after the hiatus, and a change in shopping behavior resulting in noncompliance. Those who dropped out did not differ significantly in sex, age, or assigned intervention group from the others. Twenty-two participants were excluded from the analysis due to noncompliance or missing data. The final number of participants included in this interim analysis was 64, including 19 in the control group, 25 in the 15% discount group, and 20 in the 30% discount group (Fig 1).

### Inclusion/Exclusion criteria

Screening interviews of Foodtown shoppers were conducted in-store, online, or over the phone by trained research personnel. Those who qualified were invited to a medical screening visit to obtain measured body weight, height, BMI, body composition, and blood pressure. A research study nurse drew a blood sample for analysis of fasting serum glucose (mg/dL), hemoglobin A1C (%), total cholesterol (mg/dL), HDL-cholesterol (mg/dL), LDL-cholesterol (mg/dL), and triglycerides (mg/dL). A urine sample was collected in a T-Cup^Ⓡ^ (Multi-Drug Urine Test Cup) to test for the following controlled substances: amphetamine, secobarbital, buprenorphine, oxazepam, cocaine, methylenedioxyamphetamine, methamphetamine, morphine, methadone, oxycodone, opiates, phencyclidine, propoxyphene, nortriptyline, and cannabinoids. The urine sample was also tested for pregnancy for women under age 55.

#### Inclusion

The inclusion criteria were: ages 18–70, BMI 24.5–50 kg/m^2^, accessible by phone, primary shopper of their household, shopped at Foodtown for at least 30% of groceries and committed to shopping at Foodtown for 100% of groceries during the study, and weight stable (± 5% in the past 3 months). Shoppers had to agree to the following: being randomized into one of three intervention groups, using a Foodtown loyalty card when they shopped and not sharing the loyalty card with anyone else, not planning to initiate weight loss plans by diet, medications, or programs, not eating out or ordering takeout more than five times per week, and maintaining the same smoking status for the study duration.

#### Exclusion

Candidates were excluded if they planned to participate in other related research studies or to travel for more than 6 weeks during the study, engaged in ≥ 10 hours of exercise and/or ≥8 hours of weight training per week, participated in athletic competitions such as marathons or body building, restrictive diets (e.g., vegan, paleo, ketogenic, exclusively organic, gluten free), had weight loss surgery, consumed excessive amounts of alcohol (≥ 3 drinks per day for men and ≥ 2 drinks per day for women), change in smoking patterns within the previous 3 months, current pregnancy or planning to become pregnant during the next year, given birth in the previous 12 months, currently breastfeeding or having breastfed in the previous 12 months, had a serious medical or psychiatric condition, dependent on or abusing alcohol or other drugs, or not drug-free/sober for at least 6 months, currently receiving government subsidies on food purchasing (i.e., SNAP or Special Supplemental Nutrition Program for Women, Infants, and Children (WIC)), or had a physical disability that would impact the safety of study procedures.

### Intervention

#### Price-reduction intervention

**Fig 2:**
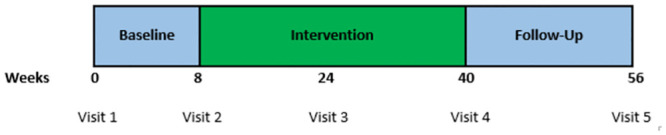
Study timeline.

Study participants were randomized by store and sex into one of the intervention groups, using *randomizer*.*org*, a web-based randomization tool, just prior to the start of the intervention period. The intervention period lasted 32 weeks, with a midpoint at week 24. At the end of the 32-week intervention, the discount was withdrawn for the subsequent 16-week follow up (Fig 2). Foods that were discounted were restricted to fresh fruits and vegetables that did not exist in mixtures with high-calorie foods (e.g., prepared salads with toppings and dressing, creamed corn, and canned fruits in syrup) and were not processed in such a way that increased their caloric density (e.g., vegetable chips and dried fruits). A list of the discounted categories were given to each participant, including those in the control group. The foods and beverages discounted included produce,.i.e., fresh and frozen fruits and vegetables, such as apples, oranges, spinach, sweet potato, canned vegetables, i.e., all single variety and mixed variety canned vegetables, such as canned chickpeas, canned corn, and non-caloric beverages, i.e., all zero calorie beverages such as bottled water, seltzer, zero-calorie sodas (excluding calorie flavored water, hot/cold coffee & tea, and regular soda). Despite the exclusion of white potatoes in a previous study [10], as they are often prepared as an energy-dense side dish, we included them here as the food item itself is low in energy-density (≤1.2 kcal/g).

A new Foodtown loyalty card (including Brooklyn Harvest supermarkets) was also given to each participant at the start of the baseline period, to be scanned at grocery checkout to deliver a point of purchase discounts on fruits, vegetables, and non-caloric beverages (as defined above).

### Measures

#### Dietary intake data

Three unannounced 24-hour dietary recalls by phone were made during the same week between visits, about 1 month before the next visit. Two weekday recalls and one weekend day recall were obtained by trained research assistants during the middle of the 8-week baseline period and about 1 month prior to the midpoint of the intervention period. The USDA five-step multiple pass method was followed during the interview to collect dietary intake data. Research staff called study participants to ask what they had to eat and drink after midnight on the previous day. During Pass 1, the researcher gathered an initial food and beverage list. Commonly missed/ forgotten foods such as beverages, snack foods, vegetables, fruits, or cheese, alcoholic beverages, and bread, rolls, or tortillas were probed during Pass 2. Information about meal times and occasions around mealtimes was gathered in Pass 3. The participant was queried about portion sizes of food and drink items during Pass 4, using a Food Amounts Booklet, which had been provided to the participant during the initial baseline visit (at Week 0), as a guide to describe the portion sizes. Finally, there was a review of the fully compiled food list with the participant during Pass 5. The three 24 h-recalls were then averaged. If there was a missing recall, the first set of dietary recalls (administered during the baseline period) and the second set of recalls (during the intervention period) were matched by weekday-weekend. For example, if a participant was missing a weekend day recall during the first set, and had all three weekday and weekend recalls completed during the second set, only the weekday recalls from the first and second set were entered for analysis.

Dietary intake data from 24-h dietary recalls were analyzed using FoodWorks 18 (The Nutrition Company, Long Valley, NJ). The nutrient report from FoodWorks 18 was used to calculate the total daily energy intake of food and beverages (kcal), energy density of solid foods (kcal/g), and total grams of: fruit and vegetable (separately and together), non-caloric beverages, i.e., diet soda (zero calorie artificially sweetened soda), bottled water, and seltzer water (separately and together), sugar-sweetened beverages, including regular soda, juice, and iced tea (separately and together), snack foods (salty and sweet, separately and together), fats/oils, and total grams of solid high ED foods (≥ 3.5 kcal/g), and solid low ED foods (≤ 1.2 kcal/g). Low ED foods generally have less fat and sugar and more fiber than high ED foods [1].

#### Clinical measures

Participant visits were at about the same time of day after an overnight fast including the end of the baseline period (Week 8) and the midpoint of the intervention period (Week 24). Clinical measures included body weight (kg) and height (cm), BMI (kg/m^2^), and percent body fat (Bioimpedance analysis; Tanita Body Composition Analyzer Model No. TBF-300A). Blood samples and systolic and diastolic blood pressure (mm Hg) were obtained by a study nurse. Whole and serum blood samples were sent to Quest Diagnostic for analysis of fasting glucose (mg/dL), hemoglobin A1C (%), total cholesterol (mg/dL), HDL-cholesterol (mg/dL), LDL-cholesterol (mg/dL), and triglycerides (mg/dL). Authors had access to information that could identify individual participants during or after data collection.

### Statistical analyses

RStudio (version 2021.09.1 Build 372) was used to analyze the data. Multiple linear regression was performed to evaluate the effects of the intervention group on the outcomes, while controlling for the outcome measures at the end of the baseline period, as well as the COVID-19 period, which occurred for 42% of participants. Additional covariates tested included sex, age, BMI, household size, number of days between measurements, annual household income, designated store, shopping percentage from designated store, race, and ethnicity. These additional variables did not have a significant effect on the dependent variables and did not change the significance of the independent variable—the intervention group, and thus were not included in the final model. We also separately analyzed the impact of the COVID-19 period. Adjusted means ± SE are shown. Two tailed *P* < .05 was considered statistically significant. Effect sizes were calculated using Cohen’s *f*^2^ where *f*^2^ = 0.02, 0.15, or 0.35 is considered small, medium, or large, respectively.

## Results

### Baseline characteristics

Baseline demographic characteristics of study participants as means and standard deviations (SD) are shown in Table 1.

**Table 1:** Baseline characteristics of the intervention groups (0%, 15%, 30% discounts) mean (SD) or n (%).

Characteristics	Mean ± SD or n (%)	Mean ± SD or n (%)	Mean ± SD or n (%)
**Age, means ± SD**	40.2 ± 11.5	39.5 ± 12.8	42.6 ± 14.6
**Household Income ($), means ± SD**	77,400 ± 56,000	57,200 ± 35,000	58,700 ± 42,200
**Sex, n (%)**			
**Female**	12(63.2)	16 (64.0)	11 (55.0)
**Male**	7 (36.8)	9 (36.0)	9 (45.0)
**Ethnicity, n (%)**			
**Hispanic**	4 (21.1)	3 (12.0)	2 (10.0)
**Non-Hispanic**	11 (57.9)	21 (84.0)	18 (90.0)
**Not provided**	4 (21.1)	1 (4.0)	0 (0.0)
**Race**			
**White**	7 (36.8)	16 (64.0)	11 (55.0)
**Black or African American**	5 (26.3)	5 (20.0)	2 (10.0)
**Asian**	2 (10.5)	0 (0.0)	2 (10.0)
**Mixed**	1 (5.3)	2 (8.0)	2 (10.0)
**Not Provided**	4 (21.1)	2 (8.0)	3 (15.0)
**COVID period, n (%)**			
**Pre-COVID**	13 (68.0)	11 (44.0)	13 (65.0)
**COVID**	6 (32.0)	14 (56.0)	7 (35.0)
**Body Weight kg), means ± SD**	80.8 ± 20.2	85.7 ± 16.6	81.4 ± 17.1
**Fat-free mass**^1^ **(kg)**	55.6 ± 12.6	56.4 ± 11.0	54.5 ± 10.0
**Fat mass**^1^ **(kg)**	25.2 ± 13.2	29.3 ± 13.7	26.9 ± 13.1
**BMI (kg/m** ^**2**^ **), means ± SD**	28.0 ± 6.6	29.3 ± 4.9	28.7 ± 5.4

^1^ Bioimpedance Analysis

^2^ SD: Standard deviation

### Fruit and vegetable intake from 24-hr dietary recalls

**Fig 3:**
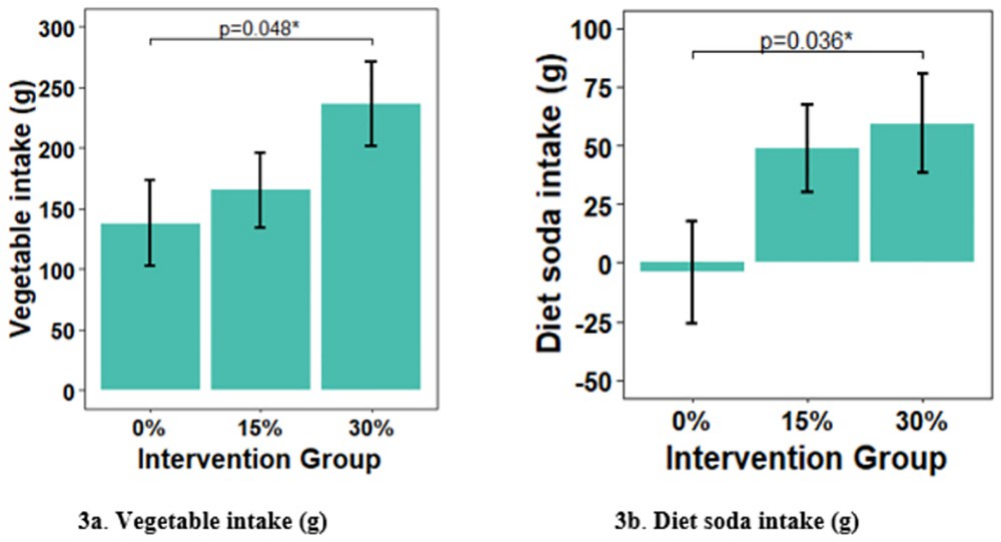
Adjusted mean values for vegetable intake (3a) and diet soda intake (3b) at the midpoint of the intervention period. Adjusted mean ± SE, *P < 0.05.

24 h-dietary intakes at the end of the baseline period and the midpoint of the intervention period are shown in Table 2. There was a significant difference in vegetable intake between the 30% and 0% discount groups, *F*(4, 59) = 2.24, t = 2.02, *P* = 0.049, *f*^*2*^ = 0.07 (Fig 3a), with the 30% group having a greater intake (+98.4 g ± 48.9) than the 0% group, accounting for a 97.2% increase from the baseline. There was no significant difference in vegetable intake between the 15% discount group and the 0% discount group, *F*(4, 59) = 2.24, t = 0.59, *P* = 0.56. There was also no significant difference in vegetable intake between the 30% and 15% discount groups, *F*(4, 59) = 2.24, t = 1.5 g, *P* = 0.15. There was no effect or interaction of the COVID period on vegetable intake.

**Table 2:** Dietary intake during the mid-intervention period by discount group.

Outcome	0%		15%		30%		15% vs 0%		30% vs 0%	
	Adj. mean	SE	Adj. mean	SE	Adj. mean	SE	t-value	P-value	t-value	P-value
**Total energy intake (kcal)**	1927	139	1881	120	1891	138	-0.25	0.80	-0.18	0.86
**Energy intake of solid foods (kcal)**	1671	125	1598	109	1727	125	-0.44	0.66	0.32	0.75
**ED of solid food intake (kcal/g)**	1.99	0.1	1.88	0.09	1.83	0.1	-0.80	0.43	-1.16	0.25
**Fruit (g)**	120.5	24.7	82.9	20.9	91.5	23.9	-1.15	0.25	-0.85	0.40
**Vegetables (g)**	138	35.1	165	30.9	236	35.2	0.59	0.56	2.02	0.049*
**Fruit and vegetables combined (g)**	259	41.2	247	35.7	327	40.2	-0.21	0.83	1.20	0.23
**Diet soda (g)**	-3.97	21.7	48.5	18.7	59.3	21.0	1.81	0.076	2.14	0.036*
**Bottled water (g)**	222	77.8	122	66.6	190	75.8	-0.98	0.33	-0.31	0.76
**Seltzer water (g)**	200.9	44.7	91.7	38.0	133.2	43.1	-1.84	0.071	-1.10	0.28
**Total noncaloric beverages (g)**	419	91.8	256	77.8	42.9	30.8	-1.34	0.19	-0.47	0.64
**Regular soda (g)**	15.85	13.0	30.87	11.2	6.63	12.6	0.87	0.39	- 0.52	0.61
**Juice (g)**	53.7	32.1	73.8	27.5	42.9	30.8	0.47	0.64	-0.25	0.81
**Iced tea (g)**	21.36	11.6	6.02	9.97	7.35	11.3	-0.99	0.32	-0.88	0.38
**Total sugar-sweetened beverages (g)**	85.1	38.4	114.4	33.2	57.3	37.0	0.57	0.57	-0.53	0.60
**Salty snack foods (g)**	21.8	6.1	20.0	5.2	11.4	5.9	-0.22	0.83	-1.24	0.22
**Sweet snack foods (g)**	95.6	17.9	81.1	14.8	81.9	9.59	-0.61	0.54	-0.55	0.58
**Fats and oils (g)**	11.2	3.06	9.37	2.6	13.12	3.0	-0.45	0.65	0.46	0.65
**High ED solid foods (g)**	115	19.7	104	16.2	130	18.4	-0.42	0.67	0.54	0.59
**Low ED solid foods (g)**	341	47.9	358	41.1	441	46.9	0.27	0.79	1.53	0.13

Adj: Adjusted; ED: Energy density

* *P* < 0.05

High energy-dense foods categorized as ≥ 3.5 kcal/g, low ED foods categorized as ≤ 1.2 kcal/g

There were no significant differences between the 15% and 30% discount groups.

Fruit intake did not differ between the 30% discount group and the 0% group, *F*(4, 59) = 1.55, t = -1.15, *P* = 0.25, or between the 15% discount group and the 0% group, *F*(4, 59) = 1.55, t = -0.85, *P* = 0.40). There was also no significant difference in fruit intake between the 15% and 30% discount groups, *F*(4, 59) = 1.55, t = 0.27 g, *P* = 0.79). There was no effect or interaction of the COVID period on fruit intake.

### Non-caloric beverage intake from 24-hr recalls

Diet soda intakes for the mid-intervention period are shown in Table 2 and Fig 3b. The 30% discount group had a significantly higher intake (+63.3 g ± 29.3) than the 0% discount group, *F(4*, *59) = 26*.*2*, t = 2.14, *P* = 0.035, *f*^*2*^ = 0.03, representing an 102.2% increase from the baseline. The 15% discount group showed a nonsignificant increase in diet soda intake (+52.0 g ± 28.6), *F(4*,*59) = 26*.*2*, t = 1.81, *P* = 0.076, compared to the control group. Diet soda intake did not differ between the two discount groups. Concomitantly, there was a decrease in regular soda intake during the intervention period in the 30% group as compared to the control group, but this decrease was not significant (-9.2 ± 17.8, *P* = 0.61). Nevertheless, the correlation for the change in diet soda vs the change in regular soda was highly significant, r = -0.67, *P* = 0001. There was no effect of the COVID period on diet soda intake, and no interaction with the intervention.

### Clinical measures

There were no significant differences in the clinical measures including body weight for the discount groups (S1 Table). However; there was a marginally significant effect of the COVID-19 period on body weight, with a relative weight gain of 1.5 kg ± 0.58 for the COVID group, *F(4*,*59) = 578*, t = 2.0, *P* = 0.053, as well as a significant increase (+1.7 kg ± 1.35) in diastolic blood pressure, *F(4*,*59) = 10*.*7*, t = 2.16, *P* = 0.035, *f*^*2*^ = 0.08.

## Discussion

The aim of the study was to determine the effects of multi-level supermarket discounts on fruits, vegetables, and non-caloric beverages on their dietary intake and on clinical markers of health. In this interim data analysis, we examined changes from the end of the baseline period to the midpoint of the intervention period. The results showed that the discounts led to significantly increased consumption of both vegetables and diet soda in the 30% discount group. The 15% discount group showed a non-significant increase in consumption of diet soda but no change for vegetables. Thus, a discount of 15% may not be adequate to influence vegetable intake. Unlike vegetable intake which increased following the 30% discount, there was no effect of the discounts on fruit intake.

Americans do not follow the dietary guidelines for fruits, i.e., adults ages 19–59 scored 2.4 out of 5 on total fruit consumption according to the 2017–2018 Healthy Eating Index [13]. The goal of the discount intervention was to encourage participants to consume more fruits and vegetables than the control group. However, the intervention did not have an impact on fruit consumption. A discount of 30% may not be adequate to alter fruit consumption. Moreover, the absence of a discount effect on fruit intake vs. vegetable intake could potentially be attributed to the diverse ways vegetables can be incorporated into an individual’s diet, as opposed to fruits which are primarily consumed during breakfast or as a snack [14]. Additionally, the shelf-life of vegetables is generally longer than that of fruits. In another study, a 50% discount did lead to an increase in fruits and vegetables [10], although that study did not analyze the effects on fruits or vegetables separately.

Although the discounts led to an increase in diet soda consumption, there was no effect on the intake of discounted bottled water and seltzer water. It is possible that in their dietary recall, participants failed to consistently distinguish bottled water from tap water. Also, seltzer water is consumed much less in comparison to diet soda, and may not have been sufficiently sensitive to the effects of the discounts.

The 30% discount led to a significant increase in diet soda. At the same time, there was a non-significant decrease in regular soda intake during the intervention period in the 30% group as compared to the control group; however, the inverse correlation between diet soda intake and regular soda intake was highly significant, indicating a strong relationship. This significant correlation suggests the increase in diet soda intake led to a decrease in regular soda intake.

There were no significant effects on body weight and body fat by the discount intervention. There was also no significant change in total daily caloric intake. A discount of 30%, or 15%, may not be adequate to produce a reduction in caloric intake or body weight. It may also take longer than 16 weeks for changes in body weight and fat to manifest following a 30% discount.

There was a marginally significant effect of the COVID-19 pandemic to increase body weight. Similarly, Lin et al. showed a significant increase in body weight as a result of the COVID-19 pandemic [15]. Factors contributing to weight gain may include, increased sedentary behavior and reduced physical activity [16], psychological stress [17, 18], and changes in dietary intake [19]. We also found a significant increase in diastolic blood pressure. We did not find an increase in energy density of solid food intake as shown previously [20].

Our results are mostly consistent with other supermarket studies. Two studies [10, 21], showed that combined intake of fruits and vegetables during an 8-week intervention period was significantly greater following a 50% discount as compared to a 0% discount control group. In the current study, only the 30% discounts for vegetables led to higher intakes. In another study [22], following a 20% discount over a 3-month intervention period, there was no effect on fruit or vegetable intake, consistent with our lack of finding an effect with a 15% discount over a 4-month period. However, a study in remote Australia showed that a 20% discount on fruits, vegetables, and low calorie bottled beverages and water did lead to a significant increase in fruit and vegetable intake over a 6-month period [23]. Thus, for a 20% discount to have an impact, it may require a ≥ 6-month period.

In the Australian study [23], there was an increase in intake of bottled water, but not diet soda, whereas we found an increase in intake of diet soda and not water. It is likely that in remote Australia, water consumption is primarily via bottled water and not from the tap, and that diet soda consumption is less than usual [24]. On the other hand, the study by Geliebter et al. (2013) did not find an increase in non-caloric beverage intake following a 50% discount over 8 weeks. This is similar to a study by Ball et al., over 3 months, in which the 20% discount intervention did not increase intake of either bottled water or low-calorie diet beverages [22]. A minimum period of 4 months may be required as in the current study to observe an effect.

In a supermarket RCT [11] of older adults (ages 40–70) in Philadelphia, the intervention group received $1 for every healthy food/beverage they purchased after submitting the store receipts and the food item labels, whereas the control group received no incentives. The intervention group significantly increased vegetable intake based on self-recorded 3-day food records, but there was no effect on fruit intake. Their results showing an increase in vegetable intake are consistent with our findings.

Due to low intake of fruits and vegetables by SNAP participants, the USDA Food and Nutrition Service began the Healthy Incentives Pilot (HIP) to test a randomly assigned 30% incentive for fruits and vegetables in Springfield, Mass. The 30% incentive was added back to the person’s Electronic Benefit Transfer (EBT) card after purchases. Daily fruit and vegetable intake for HIP participants via 24-h dietary recall was about 25% higher after 4–6 months of the program than for the control SNAP group who did not receive the incentive [12].

In the current study, we did not observe significant changes in body weight or other clinical measures. The changes in dietary intake that we observed may not be adequate to promote changes in clinical measures, such as blood pressure, lipids, and glucose, without some degree of weight loss [25–27].

### Price elasticity

Given 97.2% and 102.2% increases in vegetable intake and diet soda intake, respectively, in response to a 30% discount, the own price elasticity for intake (the absolute value of the % change in intake over the % change in price) of vegetables was 3.2, and 3.4 for diet soda. A number above 1 indicates elasticity [28]. The elasticity results apply only to the two discount groups as there is no elasticity in the control 0% group, but the change in the % intake results can be compared among all the groups.

## Strengths and limitations

Strengths in this study include the RCT protocol, use of 3-day 24-hour dietary recalls by unannounced phone calls using the USDA five-step multiple-pass method, and a relatively long intervention of 4 months, even up to the study midpoint. Additionally, the use of store loyalty cards as a means to electronically implement the discount intervention at the point-of-purchase is a novelty.

Limitations include the disruption of the study by the nationwide lockdown due to the COVID-19 pandemic, which extended the length of time between visits for many participants. However, the extended time periods did not have a significant effect on the study outcomes. Some participants did not adhere to the 100% shopping requirement at Foodtown markets. However, the percentage of total shopping at the designated supermarket also did not affect the outcome measures. Another limitation of this study may be the use of caloric density as a criteria for the discount intervention rather than the degree of food processing (not processed, minimally processed, or ultra-processed); however many dietary intervention studies use caloric density given its strong correlation with caloric intake, the main factor associated with weight change. Also, the use of self-reported measurements of dietary intake is subject to errors brought on by inaccurate reporting of food amounts and inability to recall food and drinks consumed. Finally, the large number of inclusion/exclusive eligibility criteria may limit generalizability.

In summary, the results of this study indicate that discounts, especially at the 30% level, can translate into increased intake of vegetables and diet soda. The findings show that economic incentives can promote healthful intake. Thus, reducing the price of low-ED foods, especially vegetables, can be a useful strategy for public health officials and policymakers to consider for improving access to healthful foods and beverages and promoting healthy dietary intake behaviors.

## Supporting information

S1 Table Clinical measurements at the end of the mid-intervention period for the intervention groups. There were also no significant differences between the 15% and 30% discount groups.
(DOCX)

S1 Checklist CONSORT checklist. (PDF)
