# Supplementary material for: Impact of a randomized controlled trial of discounts on fruits, vegetables, and noncaloric beverages in NYC supermarkets on food intake and health risk factors
Source: PLoS One. 2023 Nov 22;18(11):e0291770. doi: 10.1371/journal.pone.0291770 (PMC10664931; doi:10.1371/journal.pone.0291770)
Supplement: S1 Table — There were also no significant differences between the 15% and 30% discount groups. (DOCX) [file pone.0291770.s001.docx]

| **Table 1.** Clinical measurements at the end of the mid-intervention period for the intervention groups | | | | | | | |
| --- | --- | --- | --- | --- | --- | --- | --- |
|  | **Adjusted means ± SE** | | | **t-value** | **P-value** | **t-value** | **P-value** |
| **Discounts:** | **0%** | **15%** | **30 %** | **15% vs 0%** | | **30% vs 0%** | |
| **Weight (kg)** | 82.8 ± 0.68 | 83.9 ± 0.59 | 83.7 ± 0.65 | 1.29 | 0.20 | 0.86 | 0.39 |
| **Fat-free mass (kg)** | 54.4 ± 0.63 | 55.7 ± 0.54 | 55.4 ± 0.61 | 1.31 | 0.11 | 0.85 | 0.24 |
| **Fat mass (kg)** | 28.6 ± 0.83 | 28.4 ± 0.71 | 28.2 ± 0.80 | 0.75 | 0.87 | -0.14 | 0.70 |
| **Percent fat (%)** | 0.33 ± 0.0069 | 0.33 ± 0.0059 | 0.33 ± 0.0067 | 0.25 | 0.80 | -0.27 | 0.79 |
| **BMI (kg/m^2^)** | 29.2 ± 0.43 | 29.2 ± 0.37 | 28.7 ± 0.42 | 0.16 | 0.87 | -0.70 | 0.49 |
| **Systolic BP (mmHg)** | 118 ± 3.20 | 120 ± 2.74 | 119 ± 3.10 | 0.45 | 0.65 | 0.33 | 0.74 |
| **Diastolic BP (mmHg)** | 75.8 ± 1.61 | 75.2 ± 1.36 | 74.5 ± 1.55 | -0.26 | 0.80 | -0.61 | 0.55 |
| **Fasting Glucose (mg/dL)** | 91.2 ± 2.72 | 88.7 ± 2.13 | 90.2 ± 2.34 | -1.02 | 0.48 | -0.91 | 0.79 |
| **HbA1c (%)** | -0.058 ± 0.063 | -0.066 ± 0.067 | -0.060 ± 0.058 | -0.13 | 0.85 | -0.032 | 0.97 |
| **Total Cholesterol (mg/dL)** | 190 ± 5.55 | 195 ± 4.84 | 187 ± 5.30 | 0.68 | 0.53 | -0.22 | 0.62 |
| **HDL-cholesterol (mg/dL)** | 61.3 ± 1.94 | 58.3 ± 1.69 | 61.9 ± 1.88 | -1.17 | 0.25 | 0.18 | 0.81 |
| **LDL-cholesterol (mg/dL)** | 114 ± 5.68 | 116 ± 4.97 | 106 ± 5.49 | 0.36 | 0.76 | -0.78 | 0.30 |
| **Triglycerides (mg/dL)** | 93.5 ± 6.47 | 87.8 ± 5.71 | 83.1 ± 6.37 | -0.64 | 0.52 | -1.13 | 0.25 |
| There were also no significant differences between the 15% and 30% discount groups. | | | | | | |  |
